# The effects of graded levels of calorie restriction: II. Impact of short term calorie and protein restriction on circulating hormone levels, glucose homeostasis and oxidative stress in male C57BL/6 mice

**DOI:** 10.18632/oncotarget.4003

**Published:** 2015-06-01

**Authors:** Sharon E. Mitchell, Camille Delville, Penelope Konstantopedos, Jane Hurst, Davina Derous, Cara Green, Luonan Chen, Jackie J.D. Han, Yingchun Wang, Daniel E.L. Promislow, David Lusseau, Alex Douglas, John R. Speakman

**Affiliations:** ^1^ Institute of Biological and Environmental Sciences, University of Aberdeen, Aberdeen, UK; ^2^ Mammalian Behaviour & Evolution Group, Institute of Integrative Biology, University of Liverpool, Liverpool, UK; ^3^ Key laboratory of Systems Biology, Shanghai Institute of Biological Sciences, Chinese Academy of Sciences, Shanghai, China; ^4^ Key Laboratory of Computational Biology, Chinese Academy of Sciences-Max Planck Partner Institute for Computational Biology, Shanghai Institutes for Biological Sciences, Chinese Academy of Sciences, Shanghai, China; ^5^ State Key Laboratory of Molecular Developmental Biology, Institute of Genetics and Developmental Biology, Chinese Academy of Sciences, Chaoyang, Beijing, China; ^6^ Department of Pathology and Department of Biology, University of Washington, Seattle, USA

**Keywords:** calorie restriction, protein restriction, glucose homeostasis, oxidative stress, adipokines

## Abstract

Limiting food intake attenuates many of the deleterious effects of aging, impacting upon healthspan and leading to an increased lifespan. Whether it is the overall restriction of calories (calorie restriction: CR) or the incidental reduction in macronutrients such as protein (protein restriction: PR) that mediate these effects is unclear. The impact of 3 month CR or PR, (10 to 40%), on C57BL/6 mice was compared to controls fed *ad libitum*. Reductions in circulating leptin, tumor necrosis factor-α and insulin-like growth factor-1 (IGF-1) were relative to the level of CR and individually associated with morphological changes but remained unchanged following PR. Glucose tolerance and insulin sensitivity were improved following CR but not affected by PR. There was no indication that CR had an effect on oxidative damage, however CR lowered antioxidant activity. No biomarkers of oxidative stress were altered by PR. CR significantly reduced levels of major urinary proteins suggesting lowered investment in reproduction. Results here support the idea that reduced adipokine levels, improved insulin/IGF-1 signaling and reduced reproductive investment play important roles in the beneficial effects of CR while, in the short-term, attenuation of oxidative damage is not applicable. None of the positive effects were replicated with PR.

## INTRODUCTION

Calorie restriction (CR) is the most consistent intervention known to improve healthspan and delay the aging process [1–4]. Although the exact mechanisms underlying the beneficial effects of CR remain unclear, several physiological adaptations to CR are associated with an extension in longevity. First, the prevention of obesity/lowering fat mass, particularly visceral fat [5–8], reductions in glucose and insulin levels improving insulin sensitivity [9–12], a reduction in oxidative stress and/or increase in antioxidant defense mechanisms [13–16], and a reduction in body temperature [17].

A comprehensive analysis of body composition responses to graded levels of CR [18] highlighted the preferential utilization of adipose tissue, but also that reductions occur in lean tissue mass and masses of the vital organs. Adipose tissue is an efficient energy store as well as a major endocrine organ from which adipokines are derived [19, 20]. Interestingly some of the longest living mutant mouse models, Ames and Snell dwarf mice, which can live >50% longer than wild type siblings, are also relatively obese. Although these dwarf mice exhibit diminished visceral fat depots, their subcutaneous depots are larger and may be related to their improved metabolic activity [11, 21] in that while visceral fat is associated with insulin resistance, higher risk of type II diabetes, dyslipidemia, and mortality [22–24], subcutaneous fat is associated with improved insulin sensitivity, a lower risk of developing type II diabetes and as indicated by these long lived models, an extended lifespan [24–26].

Following CR, decreases in fat mass result in a concomitant reduction in fat derived hormones such as leptin, a key regulator of long-term energy balance [27]. Leptin and insulin resistance are both associated with aging and much evidence exists to show the amelioration of these metabolic disorders by CR [28–30]. The metabolic pathways controlling energy metabolism appear to be especially important in the regulation of aging and longevity [31–33]. Lean tissue is also now widely recognized as a source of endocrine factors [34]. Reductions in lean tissue mass may be expected to influence levels of interleukin-6 (IL-6) and insulin-like growth factor-1 (IGF-1) which impact on insulin disposal capability. In fact, growth hormone (GH)/IGF-1 and insulin signaling, one of the most evolutionary conserved pathways, has strong associations with aging [10]; reviewed in [35]. The long lived Ames, Snell and the GH receptor knockout dwarf mice all have reductions in GH, IGF-1 and improved insulin sensitivity [36–39]. It seems no coincidence that both the extension of lifespan mediated by CR and the long lived GH mutant mice models result in a reduced body size. Miller *et al's* 2002 paper “Big mice die young” found early life body weight can predict longevity in 4 mutant dwarf mice models [40]. On the other hand mice overexpressing GH are larger in size but with lowered adiposity and a drastically reduced lifespan compared to controls [21, 41]. Thus, while a larger body size is one of the best predictors of a longer life span across species, within species, it is the smaller animals that live longest and the larger are shorter lived [42, 43].

Ames and Snell dwarf mice also exhibit elevated antioxidant profiles, lower production of reactive oxygen species (ROS) and less oxidative damage, which are also characteristic of CR. Many have postulated that the life-extension effects of CR result from the attenuation of age-related oxidative stress [14, 16, 44–47]. However, results from these and other studies are inconsistent and exactly how these changes are facilitated by CR remains unclear. Although the free radical theory of aging (FRTA) is one of the most popular theories of aging [48–51] this has recently been called in to question [52–54].

Investment in antioxidant protection however, is potentially energetically costly therefore there is an anomaly in the upregulated response of this system in animals that are under restricted energy supply. A resolution of this anomaly is provided by evolutionary considerations of the effects of CR [55]. In particular under restriction animals may not have sufficient energy to sustain reproduction which is energetically costly [56, 57] and generally CR results in infertility but this is dependent on sex, level of CR and age [2, 58]. It is suggested therefore that under CR animals may shut down their reproductive activity and divert the saved energy towards mechanisms such as antioxidant protection that protect the soma, thereby increasing lifespan. However measurements of reproductive investment are hard to determine in mice denied access to individuals with which to mate. While reproductive effort for females is energetically expensive, particularly during lactation, for males reproductive costs include investment in territorial scent marking which can relay information regarding sex, reproductive and health status from which a female mouse can determine her selection of a mate [59]. The urine of mice contains a collection of proteins referred to as major urinary proteins (MUPs) which play an important role in scent marking [60, 61]. The production of MUPs from the liver and salivary glands together with other androgen-dependent volatile signals such as farnesenes produced in the preputial glands [59] may accrue oxidative damage. Thus, according to the trade-off theory between reproduction and somatic protection mice should alter their investment in scent marking by lowering production of urinary MUPS as they shut down reproductive process to save energy [62].

The purpose of this study was to characterize the impact of CR in the male C57BL/6 mouse on four proposed mechanisms by which a reduced food intake may alleviate the aging process. 1) a reduced level of body fat and hence inflammatory adipokines [6, 63–65], 2) alterations in the insulin/IGF-1 signaling pathways and hence improved glucose homeostasis [66, 67], 3) lowered oxidative damage and/or increased antioxidant capacity [14, 15], and 4) reduced reproductive investment (in MUPs) [58, 69]. In the majority of studies indicating a beneficial effect of CR on the life span, there was a parallel reduction in dietary protein supply, which led some to suggest the promotion of longevity is at least in part due to protein restriction (PR) [70]. Here we investigated the physiological outcome of a graded series of CR, where both protein and calories were reduced, and PR, where diets were isocaloric and the change was solely a reduction in protein.

## RESULTS

### Calorie restriction (CR)

#### Circulating hormones

**Figure 1:**
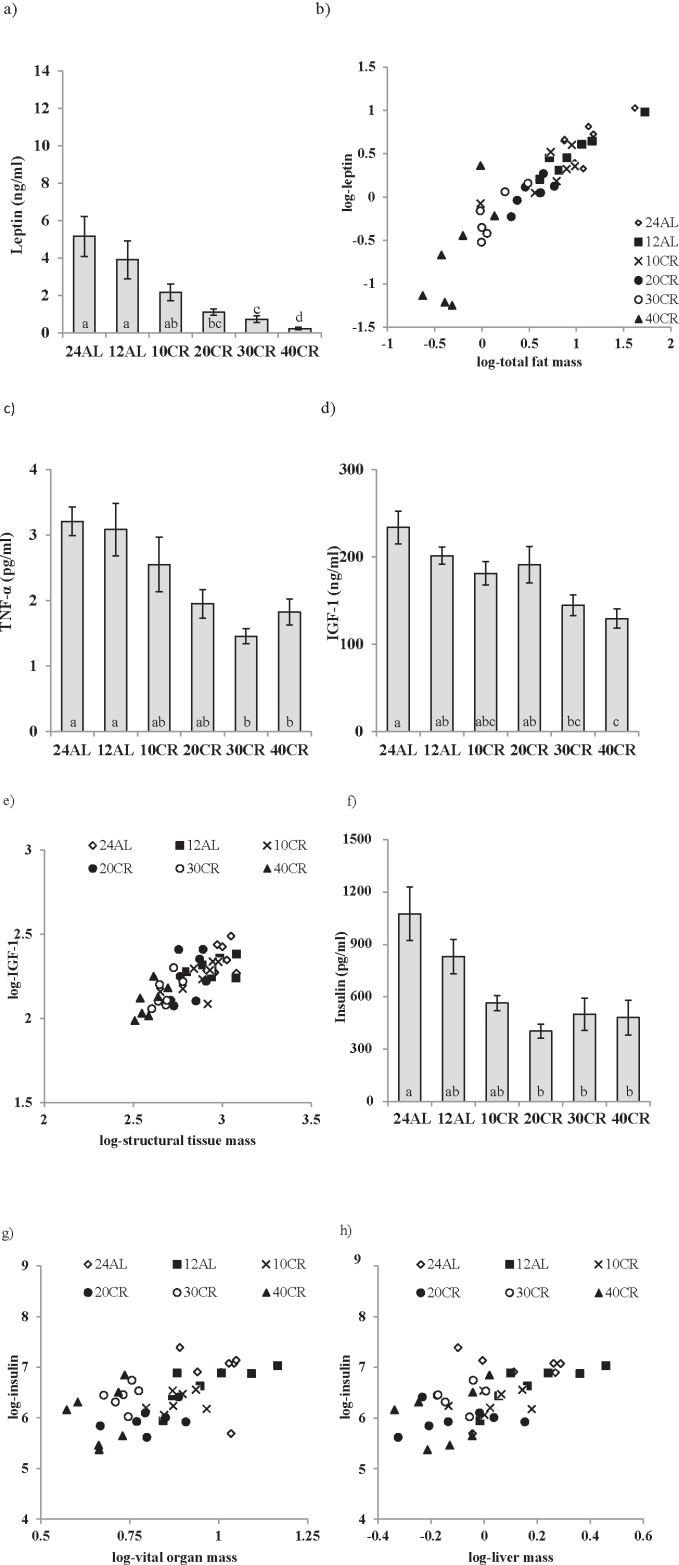
Hormonal changes measured in male C57BL/6 mice following calorie restriction (CR) Mice were fed 12 or 24 hrs *ad libitum* (12AL or 24AL) or calorie restricted (CR) by 10, 20, 30 or 40% (10CR, 20CR, 30CR and 40CR) for 3 months. Circulating levels of **a.** leptin, **c.** tumor necrosis factor (TNF)-α, **d.** insulin-like growth factor (IGF-1) and **f.** insulin. Significant hormonal relationships are shown between **b.** fat mass and leptin, **e.** structural tissues and IGF-1, **g.** vital organs and insulin and **h.** liver and insulin. Results are expressed as mean ± sem. Different letters denote significant differences between treatment groups.

A gradation in the response to CR was observed in circulating leptin levels (One way ANOVA: F_5,35_ = 14.96, *p* < 0.0005) (Figure 1a) with leptin levels lower relative to the 12AL control group (food available *ad libitum* in 12 h dark period) (3.9 ±1.02 ng/ml) in the mice where food was restricted by 20%, 30% and 40% (1.10 ± 0.16, 0.73 ± 0.16, 0.53 ± 0.28 ng/ml 20CR, 30CR and 40CR respectively, post-hoc Tukey *p* < 0.01) (Figure 1a). Consequently, level of restriction was negatively related to the circulating leptin levels (least squares regression: F_1,39_ = −79.01, *p* < 0.0005, r^2^ = 0.67). When we entered the masses of the different body compartments into a multiple regression model, along with the level of restriction, the significant predictors were the summed masses of white adipose tissue (*T* = 7.89, *p* < 0.0005: Figure 1b), structural tissue (*T* = −2.68, *p* = 0.011) and mass of the reproductive organs (*T* = 2.27, *p* = 0.029), with the overall regression explaining 84.9% of the variation in circulating leptin levels (F_3,37_ = 69.28, *p* < 0.0001). The masses of the alimentary tract and vital organs were not significant predictors (*p* > 0.05). Within the body fat compartment, circulating leptin was related to the masses of subcutaneous (*T* = 3.11, *p* = 0.004) and epididymal fat stores (*T* = 21.98, *p* = 0.056: overall regression r^2^ = 0.802, F_2,38_ = 77.03, *p* < 0.0005) but not the masses of the retroperitoneal or mesenteric fat stores (*p* > 0.05 in both cases). Within the reproductive organs the significant predictor was the mass of the reproductive accessory organs (*T* = 5.46, *p* = 0.0005) and not the mass of the testes (*p* > 0.05).

Plasma TNF-α levels (One way ANOVA: F_5,34_ = 6.15, *p* < 0.0005) were also significantly lower with 30CR and 40CR (1.45 ± 0.10 and 1.82 ± 0.17 pg/ml) when compared to 12AL (3.08 ± 0.41 pg/ml, post hoc Tukey: *p* < 0.05) (Figure 1c), and hence TNF-α was significantly negatively related to the level of restriction (least squares regression; F_1,38_ = −24.01, *p* < 0.0005, r^2^ = 0.387). None of the masses of the different body compartments entered were significant predictors of the circulating level of TNF-α. CR treatment was also a significant factor affecting levels of IL-6 (One way ANOVA: F_5,35_ = 4.75, *p* < 0.01), however, IL-6 levels were not systematically related to the level of restriction (least squares regression: F_1,39_ = 1.13, *p* = 0.29). The levels were similar in the 12AL, 10CR (10% restriction) and 30CR, and were higher in the 24AL (24 h *ad libitum* access to food), 20CR and 40CR (not shown). Nevertheless, circulating IL-6 levels were significantly associated with the total mass of the alimentary tract (multiple regression: F_1,39_ = 5.82, *p* = 0.021, r^2^ = 0.129), and more specifically the mass of the stomach (F_1,39_ = 4.93, *p* = 0.032). No association between CR level and the level of resistin was observed (F_5,35_ = 0.63, *p* > 0.05, not shown), but there was a significant relationship between circulating resistin levels and the mass of structural tissue (least squares regression: F_1,39_ = 5.32, *p* = 0.027, r^2^ = 0.119). No other tissue compartments were significantly associated to resistin levels in the multiple regression analysis.

There was a strong effect of the level of CR on plasma IGF-1 levels (One way ANOVA: F_5,38_ = 6.56, *p* < 0.0005) with significantly lower levels in the 40CR mice relative to 12AL mice (129.21 ± 9.68 versus 201.33 ± 9.06 ng/ml, post hoc Tukey: *p* < 0.05) (Figure 1d). When we entered level of restriction and the masses of the different body compartments into a multiple regression model, the circulating IGF-1 levels (logged) were strongly related to the mass of structural tissue (Regression; F_1,42_ = 47.21, *p* < 0.0005, r^2^ = 0.53: Figure 1e) but not to level of restriction, or the masses of any other body compartment. Fasted plasma insulin levels, were reduced by CR relative to 12AL (One-way ANOVA: F_5,35_ = 5.56, *p* < 0.001, Figure 1f) with levels significantly lower compared to 12AL at restriction levels of 20% and above (post hoc Tukey: *p* < 0.05). Circulating insulin was strongly related to the total mass of the vital organs (least squares regression; F_1,39_ = 16.15, *p* < 0.0005, r^2^ = 0.293: Figure 1g). Insulin was not related to the mass of any other tissue group, or level of restriction in the multiple regression model. Among the different vital organs, when entered into the model separately, the only significant factor influencing the circulating insulin levels was mass of the liver (least squares regression; F_1,39_ = 16.83, *p* < 0.0005, r^2^ = 0.314: Figure 1h).

### Intraperitoneal glucose tolerance tests (IPGTT)

**Figure 2:**
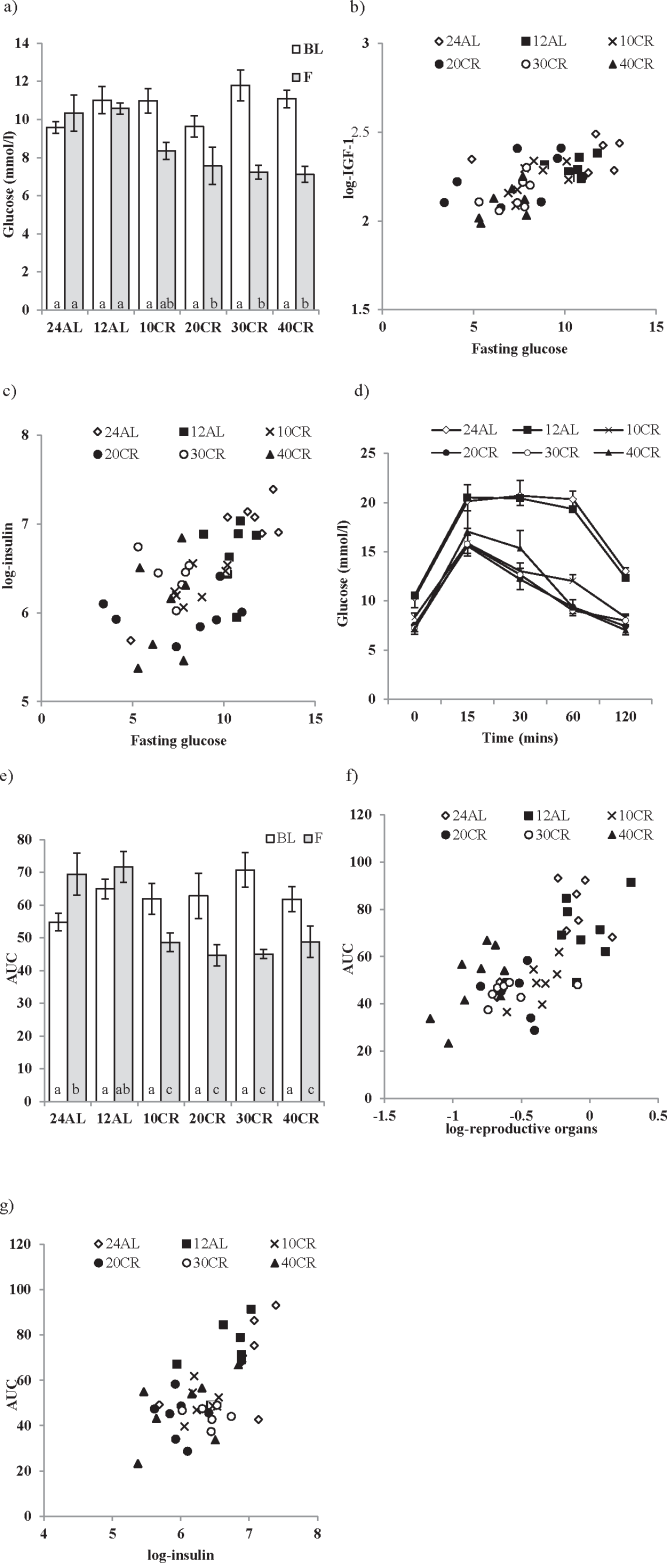
The effect of graded levels of calorie restriction (CR) on glucose homeostasis **a.** Fasting glucose concentrations at baseline (BL) and following 3 months of CR (F). Strong relationships between fasting glucose and **b.** IGF-1 and **c.** insulin were found. **d.** Glucose concentrations measured over 120 minutes following glucose injection evaluated after 3 months of CR, **e.** glucose tolerance calculated as area under the curve (AUC). **f.** Relationship between reproductive organs and AUC and **g.** insulin and AUC. Mice were fed graded levels of calorie restriction (CR) (10CR, 20CR, 30CR and 40CR) and control groups were fed *ad libitum* (AL) for 12 or 24 hrs (12AL or 24AL). Different letters within a time point indicate significant differences between groups. Data represented as mean ± sem.

There were no significant differences in fasting glucose between the 6 diet groups measured at baseline (F_5,42_ = 0.94, *p* > 0.05) (Figure 2a). Average fasting glucose at baseline was 10.28 ± 0.74 mmol/l. The level of CR was not a significant factor influencing the level of fasting glucose over the treatment period (GLM-RM, F_5,42_ = 1.725, *p* > 0.05, Figure 2a). However, there was a significant time effect when comparing baseline levels against that following 3 months treatment (GLM-RM, F_1,42_ = 49.477, *p* < 0.001) and a significant interaction between time and the level of CR (F_5,42_ = 8.269, *p* < 0.001). While fasting glucose levels between both AL groups did not differ over the 3 months (paired *t*-test, 12AL : t_7_ = 0.549, *p* > 0.05, 24AL : t_8_ = −1.818, *p* > 0.05), a significant decrease in fasting glucose was recorded in all CR groups (paired *t*-test, 10CR : t_7_ = 4.283, *p* < 0.01, 20CR : t_7_ = 2.734, *p* < 0.05, 30CR : t_6_ = 4.975, *p* < 0.01, 40CR : t_8_ = 5.213, *p* < 0.001, Figure 2a). After 3 months of treatment fasting glucose levels were significantly lowered (One way ANOVA: F_5,47_ = 7.326, *p* < 0.001) in mice under restrictions of 20CR and higher compared to 12AL (post hoc Tukey: 20CR: 7.562 ± 0.665 mmol/l, *p* < 0.001, 30CR: 7.229 ± 0.711 mmol/l, *p* < 0.05, 40CR: 7.122 ± 0.627 mmol/l, *p* < 0.01, Figure 2a). In a multiple regression analysis fasting glucose at 3 months was significantly positively related to the circulating IGF-1 levels (*T* = 4. 11, *p* < 0.0005: Figure 2b) and circulating insulin levels (*T* = 3.69, *p* < 0.001: Figure 2c), with the overall regression explaining 56.1% of the variation in fasting glucose (least squares multiple regression: F_2,38_ = 24.27, *p* < 0.0005). In this analysis fasting glucose was not related to the levels of any other measured hormones, the sizes of the different body compartments, or the level of restriction.

Glucose clearance after 15 mins was much quicker following 3 months of CR compared to 60 mins in both AL groups (Figure 2d). To evaluate this response the total area under the curve (AUC) was analyzed; a significant effect of time (GLM-RM, baseline v 3 months: F_1,42_ = 8.353, *p* < 0.001), CR level (F_5,42_ = 2.66, *p* < 0.05) and a significant interaction (F_5,42_ = 12.084, *p* < 0.001) were found (Figure 2e). Comparisons of AUC after 3 months CR (One-way ANOVA: F_5,47_ = 8.800, *p* < 0.001) to the AUC at baseline revealed an improvement in glucose tolerance with all levels of CR relative to the 12AL group (post hoc Tukey: *p* < 0.01). In contrast a negative effect of time on glucose tolerance was found in the 24AL group (AUC baseline versus 3 months, paired *t*-test, *t* = −2.557, *p* < 0.005) (Figure 2e). In the multiple regression analysis two factors emerged as significant predictors of AUC. The most significant factor was the mass of the reproductive organs (*T* = 6.84, *p* < 0.0005: Figure 2f), and in particular the mass of the reproductive accessory organs (*T* = 3.71, *p* = 0.001) rather than the mass of the testes (*p* > 0.05). The level of circulating insulin was also significantly related to the AUC (*T* = 3.00, *p* = 0.005, overall regression including both predictors F_2,38_ = 26.11, *p* < 0.0005, r^2^ = 0.579, Figure 2g).

**Figure 3:**
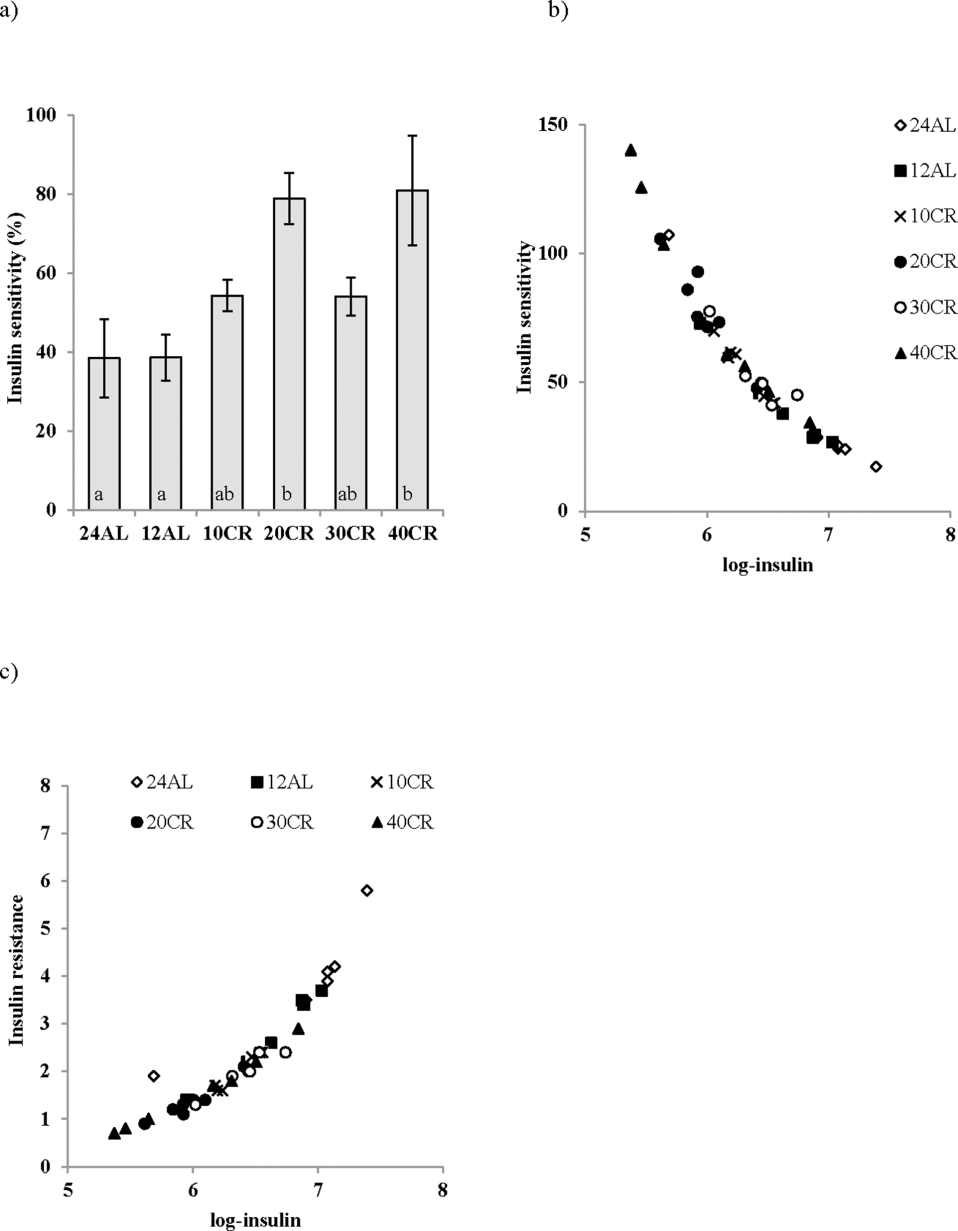
a. Improvements in insulin sensitivity following calorie restriction (CR) Insulin sensitivity was estimated from the homeostatic assessment model (HOMA2), following 3 months of graded calorie restriction (CR) (10CR, 20CR, 30CR and 40CR) or control fed 12 hr or 24 hr *ad libitum* (12AL and 24AL). Not surprisingly very strong relationships were found between insulin and **b.** insulin sensitivity and **c.** insulin resistance. Different letters indicate significant differences between groups. Results are expressed as mean ± sem.

**Figure 4:**
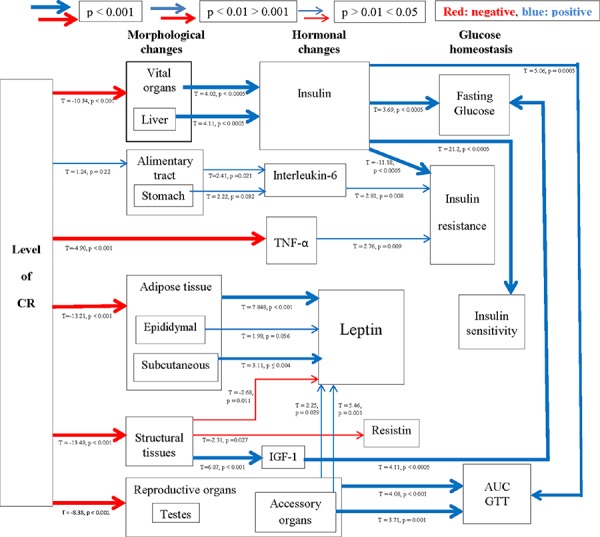
A schematic diagram depicting the highly inter-related links between morphological and hormonal changes and glucose homeostasis Positive relationships are shown in blue and negative in red, with increasing thickness of lines represent higher significance. Relationships were generated using stepwise least squared multiple regression models.

An improvement in insulin sensitivity is a key feature of the CR response. Insulin sensitivity, assessed using the Homeostasis model assessment (HOMA2), was improved by CR (One way ANOVA: F_5,39_ = 5.98, *p* < 0.001), significantly so in the 20CR (78.83 ± 6.49%, post hoc Tukey: *p* < 0.05) and 40CR groups (80.89 ± 13.87%, post hoc Tukey: *p* < 0.05) relative to 12AL (38.63 ± 5.83%) (Figure 3a). Insulin sensitivity was strongly negatively associated with variation in the circulating levels of insulin (F_1,36_ = −449.42, *p* < 0.0005, r^2^ = 0.92: Figure 3b), which was not surprising as insulin levels are part of the HOMA2 calculation (see Materials and Methods). No other factors entered as significant predictors of insulin sensitivity. HOMA2 also gives an indication of insulin resistance (One way ANOVA: F_5,39_ = 6.96, *p* < 0.001) which was significantly increased in the 20CR (1.343± 0.14, post hoc Tukey: *p* < 0.01) and the 40CR groups (1.586 ± 0.05, post hoc Tukey: *p* < 0.05) relative to the 12AL animals. As with insulin sensitivity, insulin resistance was strongly influenced by circulating insulin levels (least squares regression: F_1,39_ = 156.92, *p* < 0.0005, r^2^ = 0.878: Figure 3c). In addition insulin resistance was associated with circulating levels of TNF-α (*T* = 2.76, *p* = 0.009) and circulating levels of  IL-6 (*T* = 2.81, *p* = 0.008). The overall regression including all 3 predictors: F_3,36_ = 78.84, *p* < 0.0005, r^2^ = 0.806. These analyzes helped us infer the relationships between the level of CR, body composition changes, circulating hormone levels and parameters of glucose homeostasis (Figure 4).

### Markers of oxidative stress

**Figure 5:**
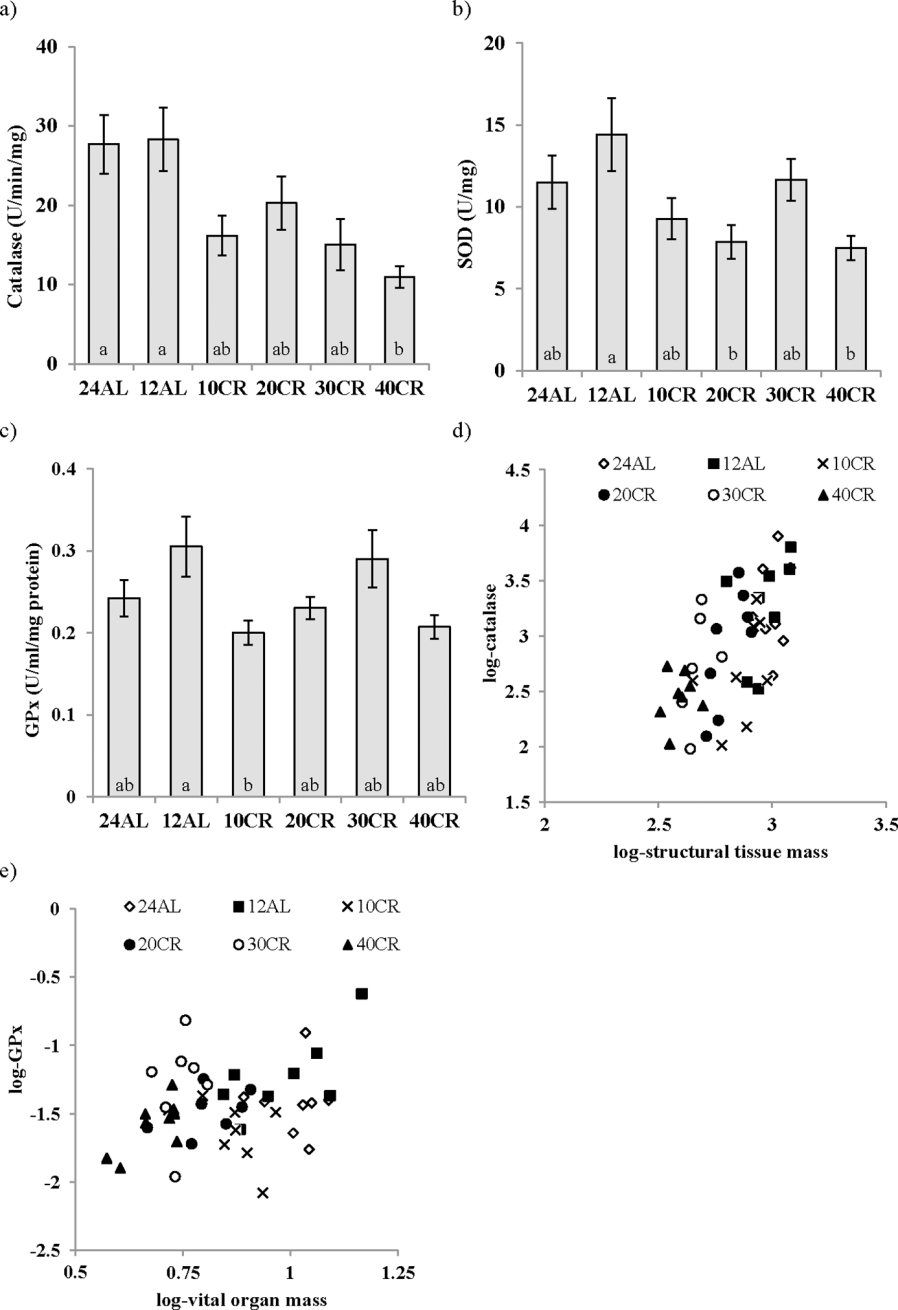
Changes in enzymatic antioxidant activity in the liver following calorie restriction (CR) **a.** catalase, **b.** superoxide dismutase (SOD), **c.** glutathione peroxidase (GPx) activity after 3 months of CR at varying levels. 10, 20, 30, 40% (10CR, 20CR, 30CR and 40CR) compared to 12 hr or 24 hr *ad libitum* feeding (12AL and 24AL). The relationships between **d.** structural tissues and catalase and **e.** vital organs and GPx are shown. Different letters signify differences between groups. Data represented as mean ± sem.

Significant variation in the levels of all three enzymatic antioxidants was observed in the liver in response to CR (Figure 5a, 5b, 5c). Catalase activity was significantly affected by the CR manipulation (One way ANOVA: F_5,48_ = 5.16, *p* < 0.001) and animals on 40CR had significantly lower levels of catalase in comparison to 12AL (post hoc Tukey: *p* < 0.01; Figure 5a). The activity of superoxide dismutase (SOD) also varied with the treatment (One way ANOVA: F_5,46_ = 3.30, *p* = 0.013) and similar to catalase was decreased following 40CR as well as 20CR relative to 12AL (post hoc Tukey: *p* < 0.05, Figure 5b). Finally, Glutathione peroxidase (GPx) levels were significantly impacted by the CR treatment (One way ANOVA: F_5,46_ = 3.17, *p* < 0.05) and were decreased significantly in the 10CR group relative to 12AL (post hoc Tukey: *p* < 0.05; Figure 5c). As with catalase and SOD, GPx activity was lowered with 40CR but did not reach significance (post hoc Tukey: *p* < 0.052; Figure 5c). Levels of all three enzymatic antioxidants were positively correlated with each other (Pearson correlation: all *p* < 0.001). The activity of catalase was associated positively with both the mass of structural tissue (*T* = 6.80, *p* < 0.0005; Figure 5d) and the mass of the alimentary tract (*T* = 3.36, *p* = 0.002): overall least squares regression F_2,45_ = 25.59, *p* < 0.0005, r^2^ = 0.532, but was not significantly linked to any of the circulating hormone levels or masses of other tissues, or the level of restriction. In contrast, activity of GPx was positively associated with the total mass of the vital organs (*T* = 3.97, *p* < 0.0005; Figure 5e) and negatively linked to both circulating TNF-α levels (*T* = −1.84, *p* = 0.044) and circulating IGF-1 levels (*T* = −3.08, *p* = 0.004): overall least squares regression F_3,36_ = 5.31, *p* = 0.004, r^2^ = 0.307. Masses of no individual vital organs were significantly associated to GPx levels (*p* > 0.08 in all cases). In the multiple regression analysis, level of restriction was a significant factor negatively influencing the levels of SOD (*T* = −2.77, *p* = 0.008) and an effect of circulating IGF-1 marginally failed to reach significance (*T* = −1.94, *p* = 0.059): overall least squares regression F_2,40_ = 3.87, *p* = 0.029, r^2^ = 0.162. The results of the OxyAdsorbent test revealed similar antioxidant power, ie oxidative defense system, between CR and AL fed mice (F_5,42_ = 0.88, *p* > 0.05). The levels of antioxidant power were not associated with any of the measured circulating hormones or masses of the different body compartments (multiple regression: all *p* > 0.05).

**Figure 6:**
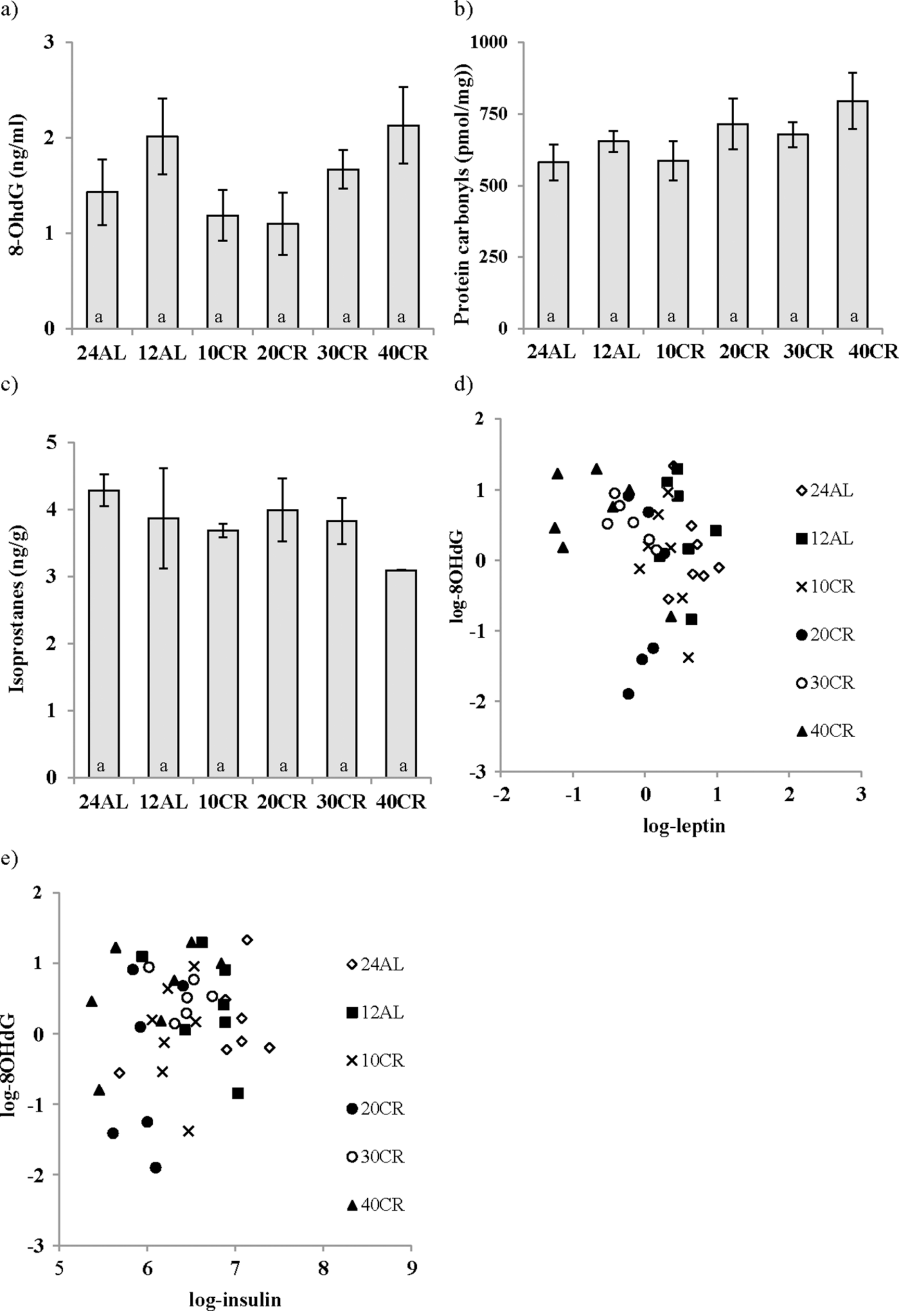
Calorie restriction (CR) did not impact changes in oxidative damage in the liver DNA, protein and lipid damage are shown as **a.** 8-Hydroxy-2′-deoxyguanosine (8-OHdG), **b.** protein carbonyls, **c.** F_2_-isoprostanes respectively. While a negative relationship between **d.** leptin levels and 8-OHdG was found e. insulin was positively linked to 8-OHdG. Mice underwent 3 months of graded CR, 10, 20, 30, 40% (10CR, 20CR, 30CR and 40CR), and were compared to 12 hr or 24 hr *ad libitum* feeding (12AL and 24AL). No differences were found between groups. Data represented as mean ± sem.

**Figure 7:**
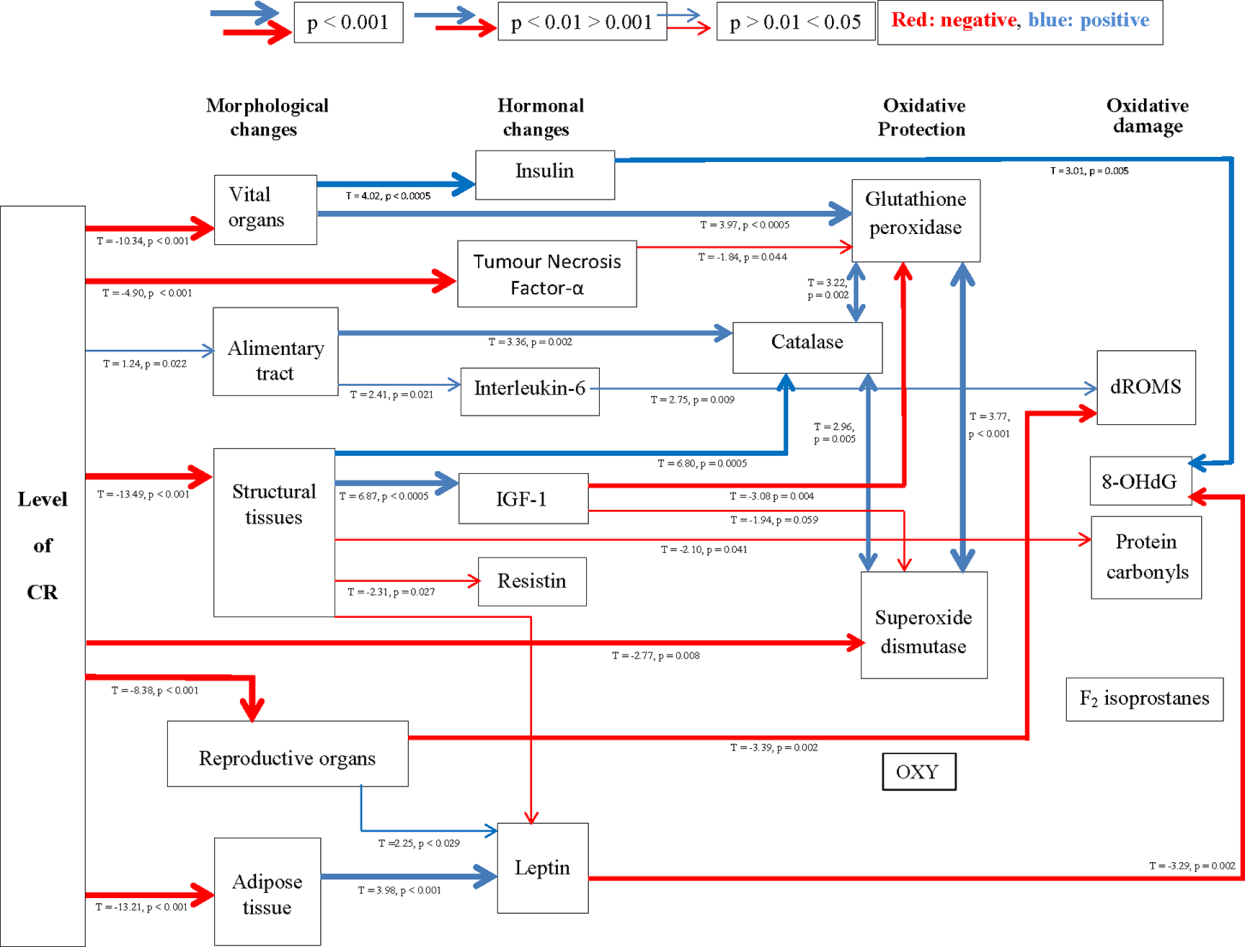
A hypothetical diagram illustrating the interactions of morphological and hormonal changes subsequently linked to biomarkers of oxidative stress Positive relationships are shown in blue and negative in red, with increasing thickness of lines represent higher significance. Significant relationships were generated using stepwise least squared multiple regression models.

The analysis of 8-Hydroxy-2′-deoxyguanosine (8-OHdG), as a marker of DNA damage in the liver, revealed no effect of CR treatment (One way ANOVA: F_5,47_ = 1.58, *p* > 0.05) (Figure 6a). Similarly liver protein carbonyl levels (protein damage) and F_2_-isoprostanes (lipid damage) were unaffected by the CR treatment (One way ANOVA: F_5,48_ = 1.64, *p* > 0.05 and F_5,17_ = 1.849 *p* > 0.05 respectively; Figures 6b and 6c). Nevertheless, levels of 8-OHdG were negatively associated with the circulating levels of leptin (*T* = −3.29, *p* = 0.002; Figure 6d) and positively associated with levels of circulating insulin (*T* = 3.01, *p* = 0.005; Figure 6e). The levels of protein carbonyls had a marginally significant negative association to the mass of structural tissue (*T* = −2.10, *p* = 0.041) but were not significantly related to masses of any other body compartment or levels of circulating hormone levels. Levels of F_2_–isoprostanes were not related to variation in any parameters of body composition or circulating hormone levels. The circulating dROMs test primarily measures hydroperoxides in the blood. A significant treatment effect on dROMs (One way ANOVA: F_5,41_ = 5.83, *p* < 0.001) suggested increased ROM production in 20, 30 and 40CR groups compared to the 12AL group (post hoc Tukey: *p* < 0.05). dROM levels were significantly positively associated with circulating levels of IL-6 (*T* = 2.75, *p* = 0.009) and negatively associated with the mass of the reproductive organs (*T* = −3.39, *p* = 0.002): overall least squares regression F_2,34_ = 12.49, *p* < 0.0005, r^2^ = 0.424. No other hormone levels, masses of tissues or the level of restriction were significant in the multiple regression analysis. None of the markers of oxidative damage (8-OHdG, protein carbonyls, F_2_-isoprostanes and dROMs) were associated significantly to levels of any of the antioxidant enzymes (catalase, SOD and GPx) or antioxidant potential (OxyAdsorbent test), and unlike the antioxidant enzymes they were also not significantly correlated with each other (Pearson correlations: *p* > 0.05 in all cases). From these results we could infer patterns of association between levels of CR, body composition, circulating hormones and markers of oxidative stress (Figure 7).

### Major urinary proteins (MUPs)

**Figure 8:**
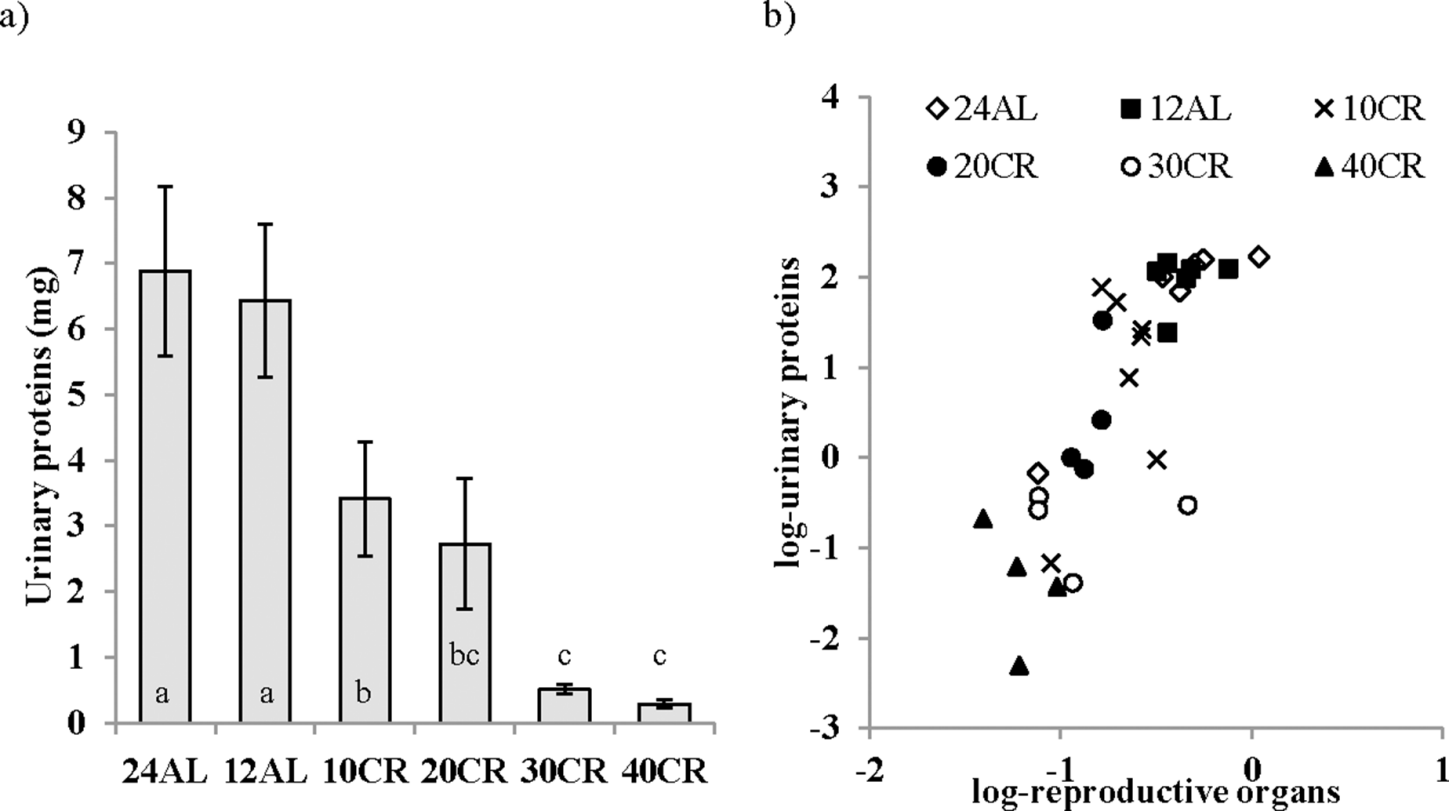
The level of major urinary proteins (MUPs) were lowered following calorie restriction (CR) MUPs measured as an indication of the level of investment in reproduction was **a.** influenced by graded CR and **b.** strongly related to changes in reproductive organs. Significant differences between groups are indicated by different letters. Data shown as mean ± sem.

The CR treatment has a significant effect on the levels of the MUPs, a marker of reproductive performance (One way ANOVA: F_5,29_ = 13.01, *p* < 0.001; Figure 8a). The level of MUPs in the urine of mice undergoing restriction at levels of 20CR and above was significantly lower than that of the 12AL group (post hoc Tukey: *p* < 0.001). MUP level was negatively related to degree of restriction (F_1,29_ = 73.33, *p* < 0.0005, r^2^ = 0.72). In the multiple regression analysis the logged levels of MUPS were significantly associated with the total mass of the reproductive organs (F_1,29_ = 53.56, *p* < 0.0005, r^2^ = 0.65; Figure 8b). This effect was due to a significant relationship with the reproductive accessory organs (*T* = 6.76, *p* < 0.0005) rather than the testes (*T* = 1.21, *p* > 0.05).

### Protein restriction (PR)

#### Hormones

**Figure 9:**
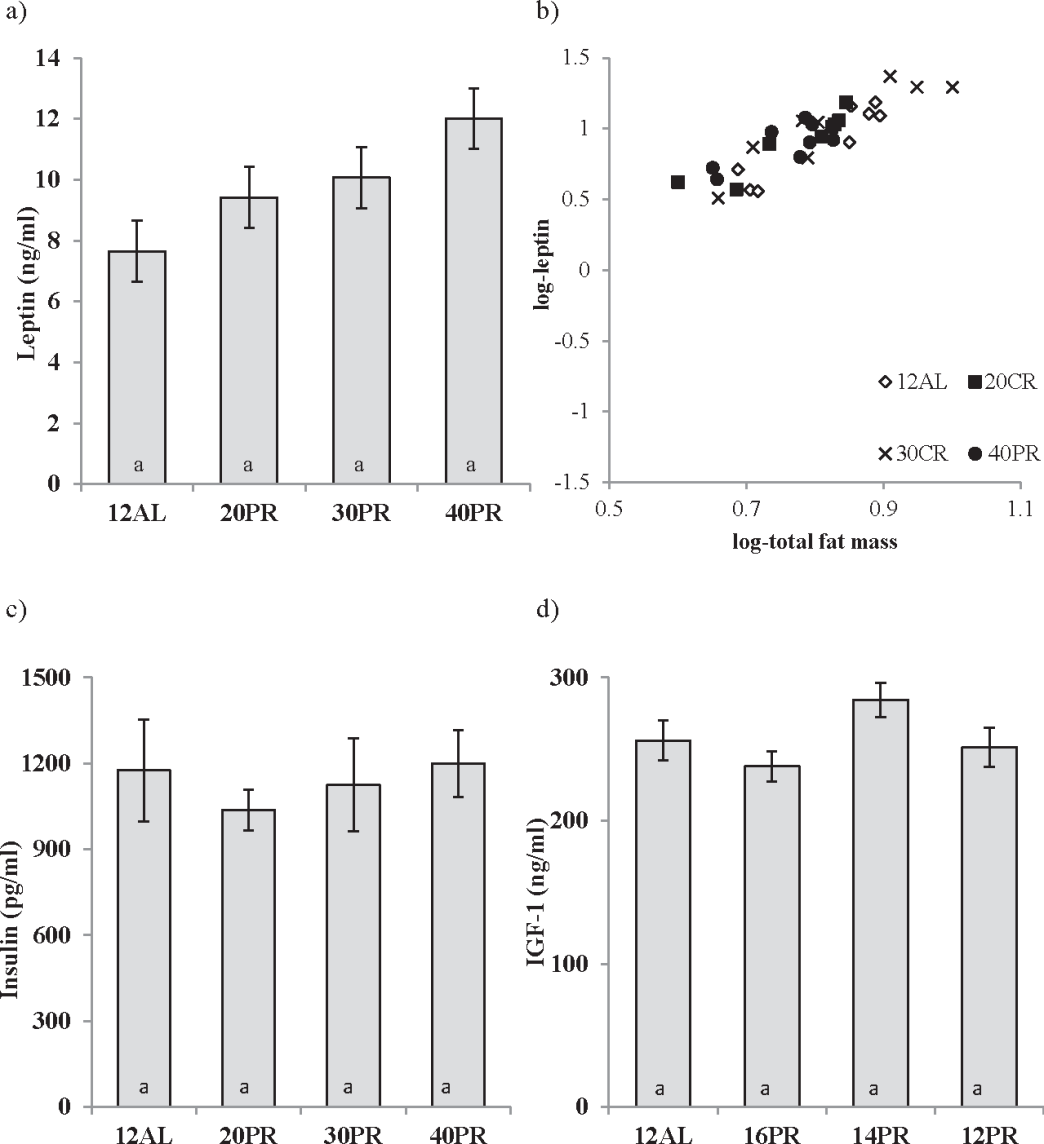
Hormonal changes measured in male C57BL/6 mice following protein restriction (PR) PR at 20, 30 and 40% of *ad libitum* (AL) intake (20, 30, 40PR) does not affect circulating levels of **a.** leptin, **c.** insulin or **d.** insulin growth factor-1 (IGF-1) measured after 3 months of PR. 12AL depicts control animals fed during 12 hours of darkness. The strong relationship between fat mass and leptin are shown in **b.** Data presented as mean ± sem. To illustrate differences between calorie restriction (CR) and PR, results are shown on the same axis as Figure 1.

No relationship between the concentration of circulating leptin and the level of dietary protein was observed (One way ANOVA: F_3,31_ = 1.022, *p* > 0.05) (Figure 9a). However, leptin levels were strongly related to the body fat mass (Figure 9b). Circulating insulin (One way ANOVA: F_3,31_ = 0.272, *p* > 0.05) and IGF-1 (One way ANOVA: F_3,31_ = 2.39, *p* > 0.05) were also unchanged by PR and at a similar level to that recorded in the AL animals (Figure 9c and 9d). Please note the same scales are used for PR and equivalent CR graphs.

### Intraperitoneal glucose tolerance tests (IPGTT)

**Figure 10:**
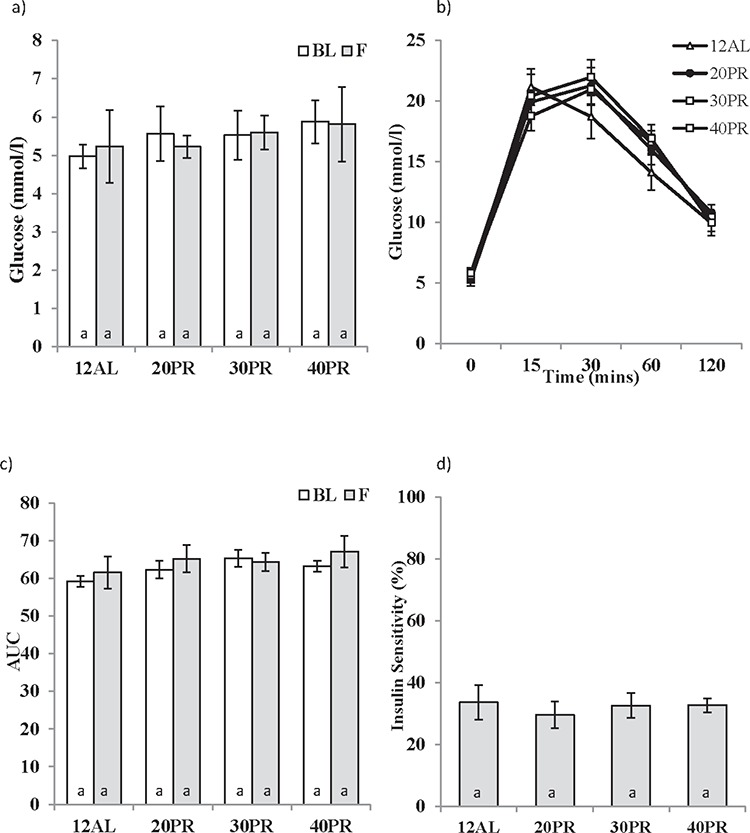
The effect of graded levels of protein restriction (PR) on glucose homeostasis **a.** Fasting glucose concentrations at baseline (BL) and following 3 months of PR (F). 20PR, 30PR and 40PR represent 20%, 30% and 40% PR. Control groups were fed *ad libitum* (AL) for 12 hrs (12AL). **b.** Glucose concentrations following glucose injection measured after 3 months graded PR, **c.** glucose tolerance calculated as area under the curve (AUC) and **d.** insulin sensitivity estimated from the homeostatic assessment model (HOMA2). Different letters within a time point indicate significant differences between groups. Data represented as mean ± sem. Results are shown on the same axis as Figure 2.

In complete contrast to the responses to CR, changes in the level of dietary protein did not affect any factors involved in the regulation of glucose homeostasis over the 3 month treatment period, ie fasting glucose (GLM-RM, Time F_1,27_ = 0.017, *p* > 0.05 and level of CR F_3,27_ = 1.236, *p* > 0.05) and AUC (GLM-RM, Time F_1,27_ = 0.863, *p* > 0.05 and level of CR F_3,27_ = 1.003, *p* > 0.05) (Figure 10a, 10b and 10c). Insulin sensitivity was also unaffected by PR (One way ANOVA, F_3,31_ = 0.600, *p* > 0.05) (Figure 10d).

### Markers of oxidative stress

**Figure 11:**
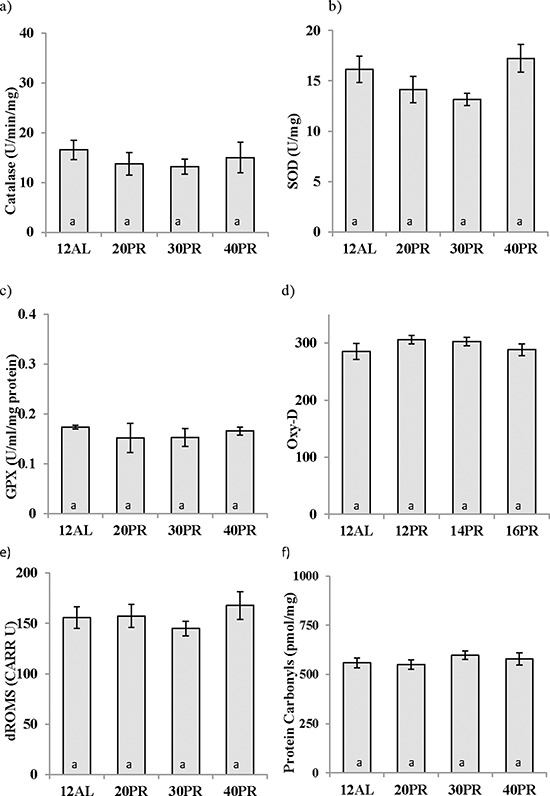
Protein restriction (PR) did not impact changes in oxidative stress biomarkers in the liver Antioxidant activity in the liver of **a.** catalase, **b.** superoxide dismutase (SOD), and **c.** glutathione peroxidase (GPx) was unchanged as was **d.** antioxidant capacity in the blood measured by OxyAdsorbent test (OxyD) following 3 months of graded PR. **e.** Protein damage as measured by protein carbonyls in the liver and **f.** reactive oxygen metabolites (dROMs) were not affected by PR reduced by 20, 30 or 40% (20PR, 30PR and 40PR) compared to 12 hr *ad libitum* (12AL) fed C57BL/6 male mice. Data displayed as mean ± sem and shown on the same axis as Figures 5 and 6 for ease of comparison of calorie restriction (CR) and PR.

None of the 3 enzymatic antioxidants measured in the liver were influenced by PR (One way ANOVA, catalase: F_3,19_ = 0.438, *p* > 0.05, SOD: F_3,19_ = 2.382, *p* > 0.05, and GPx: GPx F_3,19_ = 0.370, *p* > 0.05) (Figure 11a, 11b and 11c). Similarly no changes in the circulating levels antioxidant capacity (One way ANOVA: F_3,30_ = 1.846, *p* > 0.05) were recorded (Figure 11d). No impact of PR was noted in the production of d-ROMs (One way ANOVA: F_3,31_ = 0.370, *p* > 0.05) (Figure 11e). No indication of protein damage due to the dietary treatment, as assessed by measurement of protein carbonyls, was observed (F_3,19_ = 0.660, *p* > 0.05) (Figure 11f).

### Comparison of responses to calorie and protein restriction (CR vs PR)

To determine whether it was a reduction in calories or dietary protein that impacts the numerous health benefits reported with food restriction we drew direct comparisons between the protein matched diets, ie 40CR versus 40PR. Comparisons were first made between the 12AL groups from each study and bar two the majority of variables were statistically similar. Of the responses that did not match was fasting glucose (baseline fasting glucose: CR 11.01 ± 0.65 mmol/l vs PR 4.97 ± 0.15 mmol/l, Independent *t*-test: *t* = 9.079, *p* < 0.001 and following 3months treatment: CR 10.57± 0.29 mmol/l vs PR 5.22 ± 0.49 mmol/l, Independent *t*-test: *t* = 9.367, *p* < 0.001). These differences between PR and CR can be explained by different lengths of fasting protocols and the absolute response to glucose, as measured by AUC, were similar between the 12AL controls of the CR and PR studies (baseline AUC: CR, 64.97 ± 2.98 vs PR, 59.19 ± 1.43, Independent *t*-test: *t* = 1.745, *p* = 0.103 and following 3months treatment: CR 71.72 ± 4.70 vs PR, 61.56 ± 4.27, *t* = 1.600, *p* > 0.05). Leptin levels were also marginally different between the two 12AL groups (CR 3.9 ± 1.02 ng/ml versus PR 7.65 ± 1.34 ng/ml, Independent *t*-test: *t* = −2.168, *p* = 0.049). With the exception of protein carbonyls, comparisons of all other responses between the protein matched diets ie 20CR vs 20PR, 30CR vs 30PR and 40CR vs 40PR were significantly different, which indicates that these changes were likely driven by energy shortfall rather than the protein deficit.

## DISCUSSION

In all but a few of species [71], and some specific genetic backgrounds [72, 73], CR has been shown to alleviate age-related disease and extend lifespan. In the majority of studies examining CR, an incidental reduction in all macronutrients also occurs unless specifically compensated. One macronutrient implicated as potentially important in the CR effect is protein. Despite early research ruling out the impact of PR on the extension of lifespan, with low levels of protein apparently shortening lifespan [74–76], more recent studies have supported the notion that it is the restriction of protein that brings about the beneficial effects of CR and not the reduction in calories *per se* [77, 78]. With an increasing awareness of the importance of nutritional intervention in a number of health parameters and the more recent use of the Nutritional Geometric Framework, a state-space nutritional modelling method [79], we investigated the independent effect of CR and PR on key biomarkers of healthspan [80] ie reductions in adiposity, improvements in insulin sensitivity, a decrease in the susceptibility to oxidative damage and changes in factors closely linked to energy balance and reproduction.

Key factors affecting the response to CR are the intensity, duration and time of initiation. Increases in the intensity and length of time on CR result in a positive linear relationship with survival [3, 81] while the introduction of CR early in life can detrimentally affect development [82]. We used diets graded in the level for CR or PR, initiated at 5months of age (equivalent to early adulthood in humans), for a duration of 3 months in C57BL/6 mice. We have investigated a wealth of physiological, endocrinological, molecular and behavioral responses in these mice [18] (also Mitchell *et al* in prep).

### Calorie restriction

Although CR impacted numerous body composition changes, these effects were not mimicked when protein only was restricted and calorie intake remained at the same level [18]. Results presented here confirm this was also true for a number of hormonal and metabolic factors. These results will be discussed within the context of four popular hypotheses explaining the mechanism by which CR mediates an extension in lifespan.

### Reduction in body fat and resultant alterations in adipokine profiles

Under CR the deficit in energy must initially be attained by the utilization of tissues. We previously reported a preferential utilization of adipose tissue in these animals [18]. Over a series of studies investigating ‘Nutrition and longevity’, Berg and Simms noted that *ad libitum* feeding led to obesity in rats [63, 83]. Recognizing that the reduction in fat mass observed in the CR mice was more favorable to health and disease development they proposed that it was the reduction in body fat that mediated the beneficial effects of CR on lifespan. This theory was dismissed following observations that the lowered fat mass of restricted rats was not a factor in life-prolongation and despite being obese CR resulted in an extended lifespan in the *ob/ob* mouse [64, 65]. However, since the discovery of leptin [84] and the classification of adipose tissue as an endocrine organ, the reduction in body fat hypothesis has returned to favor [6, 7, 85, 86].

White adipose tissue serves multiple physiological functions and the reductions in adipose tissue found here under CR were strongly linked with decreases in circulating leptin levels. Leptin is produced and secreted in proportion to adipose tissue, reflecting the state of nutrition [84]. In agreement with previous reports [5] we found a preferential utilization of the visceral adipose depots (epididymal and retroperitoneal fat) as the level of CR increased [18]. Visceral adiposity is known as a major risk factor for type II diabetes, cardiovascular disease, and mortality [26, 87] and the surgical removal of visceral fat resulted in a dramatic improvement in insulin sensitivity together with a decrease in IGF binding protein-1 and a decrease in the expression of leptin and TNF-α in subcutaneous fat [26].

In the current study leptin levels were also linked to structural organs (which included carcass weight) and reproductive organ mass. This was unsurprising given the multi-functional role of leptin in the regulation of energy balance, inflammation, reproduction and bone formation [88]. Leptin is a major inhibitor of bone mass accrual [89]. Contrary to previous reports we found a beneficial effect of CR on bone mass [18] which may be related to reduction in levels of circulating leptin observed here. An association with leptin and the reproductive axis has also been well reported [90] with low levels of leptin associated with reduced fertility in both sexes. Here we also found that reduced levels of leptin were significantly associated with the levels of the Major Urinary Proteins (MUPs), a marker of reproductive investment in male mice. As a hormonal signal of available energy stores, leptin is an ideal candidate to mediate the trade-off between energy homeostasis and reproductive activity [91, 92].

In addition to leptin an ever expanding number of adipokines are produced by the adipose tissue eg TNF-α, and IL-6 are known to promote insulin resistance [96, 97] which was corroborated here. Although classed as adipokines, these inflammatory cytokines are multi-functional and here no direct relationship was found between TNF-α, IL-6 and resistin to the CR mediated decrease in fat mass. Reductions in TNF-α were dependent on the level of CR and not related to any morphological changes. With elevated levels of IL-6 associated with type II diabetes and obesity [34] we anticipated a decrease in IL-6 with CR, however this was not evident here. Unexpectedly IL-6 levels recorded here were related to the changes in the alimentary tract, specifically the stomach. The relationship between resistin and structural tissues was also unexpected and CR did not reduce resistin levels as previously reported [95]. However, overall changes found in the circulating hormones measured here following 3 months of CR were consistent with morphological changes. The gradation in CR intensity imposed the same manner of gradation in the circulating levels of leptin which may qualify leptin as a potential modulator of the anti-aging effects of CR as previously suggested [96, 97].

### Insulin/insulin-like growth factor-1 (IGF-1) signaling pathway (IIS)

Here we found reductions in plasma insulin and IGF-1 following CR, strongly impacted on fasting glucose, with insulin also influencing insulin sensitivity and insulin resistance. Glucose tolerance, as measured by area under the curve (AUC) was significantly improved with CR, and unsurprisingly related to insulin. AUC was also related to reproductive organs, specifically the accessory organs. Enhanced insulin sensitivity is a key determinant of healthy aging thought to represent an important mechanism behind CR mediated longevity and is also characteristic of many of the long-lived mutant mice [36–39]. Contradicting the hypothesis that an improvement in insulin sensitivity is responsible for enhanced longevity are the insulin receptor substrate-1 (IRS-1) knockout and Klotho mice [98, 99]. IRS-1 knockout females are longer lived with higher circulating insulin and impaired glucose tolerance in comparison to controls. While deletion of the Klotho gene resulted in reduced lifespan, overexpression significantly increased lifespan [98, 100]. However Klotho transgenic mice are IGF-1 and insulin resistant with elevated levels of IGF-1 and insulin.

One of the most popular pathways implicated in the CR-mediated increase in lifespan, is the nutrient-sensing pathway insulin/IGF-1 signaling pathway (IIS). A wealth of literature supports the notion that alterations in this evolutionarily conserved pathway can increase life span [101]. This has been demonstrated following the ablation of insulin producing cells, IGF-1, and their receptors in mice [35, 99, 102–104]. IGF-I is essential for growth and development which agrees with results found here, with IGF-1 strongly linked to the structural tissue mass. IGF-I has also been implicated as a major risk factor for the development of several different types of cancer. The reduced risk of age-related diseases, particularly cancer is associated with CR which could therefore be related to the reduced IGF-1. The administration of IGF-I *in vivo* increases the growth of tumors while decreases in IGF-1 levels results in a reduction of tumor growth [101, 105, 106].

Another means by which IIS may regulate lifespan is via a reduction of oxidative stress. This is clearly shown in C. elegans, where all single gene mutants of the nematode (>40) displaying increased lifespan had an improved resistance to oxidative stress [107]. While in rodents, the long lived dwarf mice, Ames and Snell as well as IGF-1R^+/–^ knockouts, all with an altered IIS, display stronger resistance to toxic attack from diaquat or paraquat [103, 108, 109]. Stronger resistance implies an increased defense system which agrees with results here with IGF-1 negatively impacting upon GPx and SOD activity in the liver, ie IGF-1 levels lowered via reduced structural tissue under CR leading to increased antioxidant activity (Figure 7).

The matching gradation found in response to the graded levels of CR is strongly supportive that lowered insulin and glucose, improvements in insulin sensitivity, together with reductions in IGF-1 are key to the anti-aging and life-prolonging effects of CR. However, although centenarians have low levels of IGF-1, it should be noted that the reduction in IGF-1 levels may not be applicable to humans, with short-term (6 days) [110] but not long-term (6 years) CR reducing circulating levels IGF-1 [111].

### Attenuation of oxidative damage

Contrary to previous reports [15] we found a comprehensive decrease in the activity of the enzymatic antioxidants, superoxide scavenger, SOD, and the H_2_O_2_ reducers’ catalase and GPx in the livers of mice following 3 months CR. It is unclear whether antioxidant defense mechanisms are inducible or constitutive, with literature in support of both. Until recently evidence suggested antioxidants were inducible with the level of antioxidants increased in response to low level radiation [112], or the activation induced by H_2_O_2_ [113]. However employing microarrays, which comprised all known antioxidants Desaint and colleagues found no evidence of antioxidant regulation by H_2_O_2_ and concluded mammalian antioxidant defenses are constitutive [114]. Our data do not support the suggestion that these antioxidants work in a compensatory manner [115] ie as one increases the others decrease, with levels of all three antioxidants found to correlate strongly and positively. The oxidative stress theory predicts that the action of CR reduces damage from ROS through the activation of antioxidants [48, 49] but also refer to [52–54]; results here oppose this notion, but are in agreement with a study in non- and diabetic rats also undergoing a short, 9 weeks, restriction at 40CR [116]. Although the antioxidants were strongly correlated to each other, interestingly they were individually related to separate morphological or hormonal changes. The link with IGF-1 gene deletion and increased resistance to oxidative stress was mentioned earlier and was corroborated here with a negative relationship between IGF-1 and SOD and, together with circulating TNF-α, GPx activity. Catalase and GPx were also linked independently to morphological changes resulting from the CR treatment. Catalase levels were associated positively to both the alimentary tract and structural organ masses. Despite the fact these antioxidants were measured in the liver, the weight of which was included in the vital organs, only GPx levels were positively associated to these organs. However our results suggest a mismatch of the oxidative defense system in liver compared to blood, in that antioxidant capacity (measured by OxyD) was unchanged following CR and unrelated hormonally or morphologically.

Obesity has been linked to the diminished activity of antioxidants [117] and also high oxidative stress [118, 119], if the opposite was to be true and fitting with the FRTA then following CR we would anticipate an increase in antioxidants (negative) and decrease in oxidative stress (positive relationship). As reported above antioxidant activity was lowered here. Regarding markers of oxidative stress only 2 direct relationships with morphological changes and oxidative damage were found; firstly, structural tissues and protein damage and secondly reproductive organs and dROMs. With the high protein content of muscle a connection with protein damage, as measured by protein carbonyls, may be anticipated; however both these relationships were negative therefore we again dismiss the attenuation of oxidative stress theory.

A positive relationships linking CR to oxidative stress was found between IL-6 and dROMs. In addition a positive relationship between insulin and the DNA damage marker (8OHdG) was noted however this was opposed by a negative link to leptin levels. In support of our findings, insulin was shown to induce free radicals production [120] and reverse the lowering of ROS production via CR [121]. Lambert *et al* suggested lowered insulin levels and alterations in insulin signaling may be important to lower the production of ROS [121]. The negative effect of leptin on DNA damage is unclear but leptin was previously shown to directly stimulate the production of ROS [122, 123]. However, like the antioxidants some conflicting reports exist [124]. In contrast to our results the majority of studies indicated a reduction by CR in the age-related increase in oxidative damage to DNA [125]. Sohal's 1994 study clearly demonstrated an age-related increase in 8-OHdG in skeletal muscle, brain, heart, liver and kidney and the concentration of 8-OHdG, in all 5 tissues, was reduced by 40% CR compared to *ad libitum* fed mice [13]. However it should be noted that the duration of CR in the study of Sohal was 15months thus the lack of effect noted here may merely be an indication of our short term study [13].

The age related increase in the level of protein carbonylation and F_2_-isoprostanes, markers of damage to proteins and membrane phospholipids, in the liver and kidney was previously shown to be reduced by CR [126, 127]. However, here no effect at any level of CR was found on the levels of liver protein carbonyls or F_2_-isoprostanes which again may be related to the short-term, 3 months, exposure to CR. However the lack of oxidative damage found in the liver was opposed with increased levels of dROMs measured in the blood, an indication of increased oxidative stress. It should be stressed that the measurement of oxidative damage is critically dependent on the biomarker measured and the tissues used [128, 129].

The decreased activity of antioxidants found here could be interpreted as reflecting a reduced state of oxidative stress ie less antioxidants are produced as only low levels of ROS are present, thus maintaining levels of damage constant. Reducing antioxidants in the face of less damage might conserve energy. However this does not fully correspond with our results as no correlations were evident between the antioxidants and damage to DNA, protein or lipid samples from liver. Although we cannot conclude on CR effect on lifespan extension, overall results found here do not support the attenuation of oxidative stress theory as a fundamental mechanism impacting the beneficial effects of short-term CR.

### Reproductive trade-off

CR is known to reduce fertility, a response that some have hypothesized to have evolved allowing animals to survive periods of food scarcity by shutting down reproduction and investing all available energy into survival [62, 69]. To compensate for energy restriction animals must withdraw energy from body tissues (detailed elsewhere [18]). We have previously shown that although the testes were included in tissues ‘protected’ following CR, the reproductive accessory organs were one of the preferentially utilized tissues. These included the preputial glands where farnesenes, which are linked to male sexual signaling, are produced [59]. Also important are MUPs, involatile proteinaceous scent components found in urine. For males, scent marking to attract females comes at a cost, with substantial investment in protein synthesis and resultant oxidative damage. However mixed results in house mice found that although increased oxidative damage was measured in the muscle of males under heightened stress of breeding in the vicinity of competitors, higher oxidative damage in the liver and serum was measured in males under no such stress [130]. Here the decrease in accessory organ mass together with the reduction in MUPs indicated a decrease in scent marking activity under CR and hence reduced reproductive investment. This was consistent with the suggestion that diversion of energy away from reproduction towards somatic protection is a key factor involved in the life enhancing effects of CR. These changes in MUPs were mostly closely linked to the size of the accessory organs, size which unlike testes declined under increased levels of CR [18].

### Protein restriction (PR)

Although a number of studies have reported increases in maximum longevity following PR [82, 131], here we found no changes in any of the parameters we measured that have been previously implicated in the longevity effects of CR. Improvements in insulin sensitivity is a key characteristic of CR that was not replicated by PR. The lack of response in the levels of IGF-1 and adipokines were unsurprising given the body composition of PR mice remained unchanged [18]. One of the most highly reported ideas behind the life extending effects of PR is the attenuation of oxidative stress. PR has been shown to exert life extending effects through a decrease in the accumulation of oxidative damage and increase in antioxidant defenses [126, 132, 133] (also see recent review [134]), however no such changes were found here. In fact the accumulation of oxidatively damaged proteins was found to be of a similar magnitude in mice fed the PR diets compared to mice fed the protein matched CR diets.

In addition to PR, restriction of the essential amino acid methionine (MR) has also been reported to enhance longevity, potentially mediated via impacts such as lowered serum glucose, insulin and IGF-I plus an increased resistance to oxidative damage [132, 135, 136]. Interestingly, unlike PR, changes in body composition, particularly reduced visceral fat, accompanied MR with subsequent reduction in insulin, glucose and leptin [137]. Although some believe MR to play a contributory role in CR effects, comparisons of gene expression profiles do not significantly overlap, suggesting independent pathways between CR and MR [138].

Why our results do not encapsulate those previously shown following PR is unclear. The restricted diets in this study were specifically formulated to match the level of protein used in our CR study (16, 14 and 12% protein). There is a possibility the level of PR was not stringent enough as studies where PR has shown a life extending effect the diet contained a range from ∼5 to 15%, protein, although the levels used here were at the top end of the range they were still within the range [131, 139]. However, consistent over the studies was that in the cases where PR did extend lifespan, this was to a lower extent of that demonstrated by CR, with PR only accounting for ∼50% of the life-extending effect of CR [136]. It should be noted that studies looking at the response to reductions in dietary fat [140] or carbohydrate [141] without an overall energy deficit also do not replicate the beneficial effects related to CR.

## CONCLUSIONS

None of the four theories emerged as solely explaining the beneficial effects of CR. Instead, we identified a highly inter-related network of responses supporting three of the four theories: i) body fat reduction, ii) modulation of insulin sensitivity and the IIS pathway, and iii) reproductive investment trade-offs. CR influenced hormonal changes; in particular reductions in leptin and TNF-α. Both these hormones were reduced in a graded manner relative to the level of CR with leptin specifically linked to reductions in fat mass, structural tissues and reproductive organs. How the reduction in leptin might contribute to the aging process is not fully understood. We know that disruption of leptin signaling and leptin resistance plays a critical role in obesity, a major risk factor for morbidity and mortality, and obesity-related disease such as impaired glucose metabolism, cancer, and the development of cardiovascular disease. Improvements in insulin sensitivity and modulation of the IIS pathway and the subsequent increase in lifespan are much better understood with pathways conserved from worms to humans [142]. The hypothesis that CR decreases oxidative damage and increases antioxidant enzyme levels was not supported here. Differences between studies, duration, diet composition and severity of CR are all important factors which need to be taken into account, as well as tissues and assays used. Our results do however unequivocally show that, in the short-term, 3-month PR does not replicate the changes observed with CR and this may be attributed to the lack of change in body composition changes under PR.

## MATERIALS AND METHODS

### Animals

All procedures were reviewed and approved by University of Aberdeen ethical approval committee and carried out under the Animals (Scientific Procedures) Act 1986. A detailed description of the experimental procedures has previously been published [18]. Briefly, male C57BL/6 mice (Charles River, Ormiston, UK) were individually housed on a 12:12 h light/dark cycle with free access to water and food (D12450B, Research Diets, NJ, USA). Body mass (BM) and food intake (FI) were recorded daily, immediately prior to feeding. Full details of the body composition responses are reported elsewhere [18]. To overcome obesity issues typical of *ad libitum* (AL) fed control groups with 24 hr access to food (24AL) [143], an additional control group (0%CR) fed only in the 12 hrs of darkness (12AL) was introduced, ie mice were fed at 1830 and food was removed at 0630. This not only counteracted overeating, but also ensured mice were in a similar hunger state at the time of kill, ∼4 hrs prior to lights out.

Following a 2 week baseline period, a 3 month restriction period (CR or PR) was initiated at 5 months of age, approximately equivalent to human early adulthood. The 3 month restriction period was equivalent to around a tenth of their lifespan, comparable to ∼8 years for humans. For the CR study mice were randomly allocated into the following groups (*n* = 7–9): 0, 10, 20, 30 or 40% CR (termed 12AL, 10CR, 20CR 30CR and 40CR). Restricted FI's were calculated based on individual FI over the baseline period. Mice on the CR study were fed D12450B, 20% protein, 70% carbohydrate and 10% fat (by energy), throughout. We found that mice responded to restriction by enlarging their alimentary tracts and increasing digestive efficiency [18] and hence realized levels of restriction were slightly lower than the nominal levels. All mice on the PR study received D12450B over the baseline period. This was replaced by modified diets during the restriction period, where protein content was reduced to 16, 14 and 12% matching the protein levels in the 20, 30 and 40CR groups without a reduction in calories, termed 20PR, 30PR and 40PR, respectively (D13020201, D13020202 and D13020203 respectively, Research Diets, NJ, USA). Mice in the PR study were prevented from overeating by feeding a fixed level of food daily based on their individual baseline intake. Control mice in the PR study were fed the D12450B throughout ie 20% protein, but again restricted to the hours of darkness.

At 8 months of age, after 3 months restricted feeding, the mice were killed by a terminal CO_2_ overdose. Cardiac puncture blood samples were divided between Heparin and EDTA treated tubes (BD Bioscience, UK) and later centrifuged for plasma collection. All remaining tissues were rapidly removed (∼8 mins) weighed, divided appropriately for future analysis and snap frozen in liquid nitrogen. The liver was quickly divided into 7 pieces and individually frozen in cryovials to avoid freeze/thaw artefacts.

### Plasma assays

To avoid the level of hunger affecting the concentration of adipokines and hormones measured, all mice were fasted prior to kill. Food was removed from both the 12 and 24AL groups at 0630 on the day prior to the kill at ∼1400–1700 h. Circulating insulin, leptin, tumor necrosis factor (TNF)-α, resistin and interleukin (IL)-6 were measured in plasma at the end of 3 month restriction period using the Milliplex^TM^ mouse adipokine panel (MADPK-71K, Millipore, Watford, UK). Fasted plasma IGF-1 was detected using a mouse specific Enzyme Linked Immunoassay (ELISA) (R&D Systems Europe Ltd, Abingdon, UK).

### Intraperitoneal glucose tolerance tests (IPGTT) and the homeostatic model assessment (HOMA2)

IPGTT were performed twice, firstly during baseline then after ∼11 weeks of restriction. Mice were fasted for at least 8 hours prior to IPGTT. Fasted glucose was measured from tail blood immediately prior to a 2 mg/g intraperitoneal glucose injection. Following injection glucose was measured at 15, 30, 60 and 120 minutes using Johnson and Johnson's OneTouch^®^ Ultra Blood Glucose Monitoring System. Insulin sensitivity and insulin resistance were evaluated according to the HOMA2 Calculator^©^ (The University of Oxford) (fasting blood glucose (mmol/L) × fasting insulin (μU/ml)/22.5) using fasting glucose measurements taken immediately prior to kill.

### Biomarkers of oxidative damage

As a measure of oxidative stress DNA, lipid and protein damage was evaluated in the liver along with the activity of antioxidants, catalase, glutathione peroxidase (GPx) and superoxide dismutase (SOD) in the blood. To note, in the PR study only 5 liver samples from each group were analyzed. Protocols are briefly described below.

8-Hydroxy-2′-deoxyguanosine (8-OHdG) levels, widely used as biomarker of oxidative DNA damage, were measured using the Highly Sensitive Competitive ELISA kit (Japan Institute for the Control of Aging (JAICA), Shizuoka, Japan). The intra-assay and inter-assay variations, as reported by the manufacturer, were 1.4–2.1% and 1.9–7.1%. To decrease the risk of artificial DNA oxidation, the extraction process was optimized to include sodium iodide and enzymatic DNA digestion [144]. Protein Carbonyls were measured by ELISA (BioCell, Papatoetoe, New Zealand) [145]. The measurement of liver F_2_-isoprostanes was carried out at the Barshop Institute for Longevity and Aging Studies at the University of Texas Health Science Center at San Antonio under the kind supervision of Wenbo Qi in the lab of Holly Van Remmen. The process involves lipid extraction, thin layer chromatography (TLC) purification, and quantification by gas chromatography-mass spectrometric analysis and is explained in full detail elsewhere [127]. Antioxidant enzyme activities were measured using direct spectrophotometric methods. First homogenates from ∼50 mg of liver were prepared in ice-cold 50 mM phosphate buffer (PB) (pH7.4), centrifuged and supernatants collected. All reactions were carried out in triplicate and absorbance read at 25°C using the SpectraMax Plus^384^ spectrophotometer with SoftMax software. Catalase activity was measured on the day of homogenization following the methodology of Cohen [150]. Incubation with 1% Triton X ensured the release of catalase activity from the peroxisomes. Samples were run alongside blank (buffer) and standard (water) reactions. 6 mM hydrogen peroxide (H_2_O_2_) was added to sample and blank followed by 2 mM potassium permanganate to all. Reactions were stopped after 3 min by the addition of 3 M sulphuric acid. The residual potassium permanganate color from the catalase driven peroxidation was measured at 480 nm at 25°C. Glutathione peroxidase (GPx) activity was determined by a modification of the method of Paglia and Valentine [151]. Reaction mixtures consisted of 1 mM Ethylenediaminetetraacetic acid (EDTA), 4 mM sodium azide, 0.2 mM Nicotinamide adenine dinucleotide 2-phosphate reduced tetrasodium salt hydrate (NADPH), 1.083 U/ml glutathione reductase and 4 mM reduced glutathione in ice cold 50 mM PB. Reactions were initiated by the addition of H_2_O_2_ and monitored for 1min at 340 nm. Absorbances were recorded in a quartz cuvette at 340 nm and activity calculated from the oxidation rate of NADPH to NADP. One GPx unit is directly proportional to the amount of NADPH consumed in nmol per minute at 23–25°C. Total superoxide dismutase (SOD) activity was measured using a method originally described by [148]. The assay is based on the rapid inhibition of the auto-oxidation of pyrogallol (1,2,3-benzenetriol) by SOD. Reactions were performed in a 50 mM Trizma buffer containing 1 M of the iron chelator diethylenetriaminepentaacetic acid (DPTA). The reaction rate, in the presence and absence of sample, was measured as the change in absorbance at 420 nm over a 2 minute interval. This method cannot determine between the different forms of SOD, ie CuZn, Mn and FE SODs. Specific antioxidant activities were calculated on the basis of concentration of protein per assay. Protein content was detected using the Bradford assay [149]. Standard curves were created from known concentrations of bovine serum albumin (BSA). Briefly Bradford Reagent Working solution (BioRad, Hemel Hempstead, UK) was added to 10 μl of sample, in a 96 well plate, and absorbance read at 595 nM. Plasma antioxidant potential and reactive oxygen metabolites (ROM) were evaluated in plasma samples using the OXY-Adsorbent (OxyD) and d-ROM kits respectively (Diacron international, Grossetto, Italy). Antioxidant power is expressed as μmol of HCIO/ml and ROMs, primarily hydroperoxides, results are expressed as Carratelli Units (CARR U).

### Major urinary proteins (MUPs)

Urine was collected immediately prior to kill by scruffing the mice over a Petri dish and gently massaging the bladder. Urine samples were transferred to eppendorfs and stored at −20°C. MUPs were measured using methods previously described in detail elsewhere [130, 150]. Briefly, the concentration of urinary protein was assessed using the Coomassie plus^®^ protein assay reagent kit (Perbio Science, UK). Samples were then analyzed by SDS-PAGE gels to confirm that urinary protein consisted of MUPs rather than other proteins that had leaked through the glomerular filter [134].

### Statistical analyses

Statistical analyses were performed using the PASW Statistics package 18, Minitab 16 and the R statistical environment. Data was first checked for normality using the Kolmogorov-Smirnov test and log transformed where appropriate. One way ANOVA was used for comparison between groups following the 3 month diet treatment. A general linear model with repeated measures (GLM-RM) was used to compare data across the restriction period, ie baseline versus following 3 months of CR (F). Paired *t*-tests were used where appropriate and post hoc Tukey tests compared differences between the 6 diet groups at specified time points. We have shown previously [18] that graded CR results in a cascade of effects on the sizes of different body compartments, including adipose tissue, structural tissues such as skeletal muscle, reproductive organs and to a lesser extent the vital organs. Using these body composition data, from the same animals, measured after 3 months of restriction, we sought to explore the roles of these downstream effects on body composition on the resultant changes in the levels of circulating hormones. To do this we included the level of restriction (as a continuous variable) and the masses of the different body compartments (log transformed as necessary) as independent predictors in stepwise least squared multiple regression models, with sequential backward deletion. We did not force the models by default to include any parameters. When we found a significant effect of an overall body compartment (e.g. white adipose tissue) we performed further analyses including as predictors the components that contributed to the total weight of that tissue compartment (for example, in the case of white adipose tissue including the mesenteric fat, retroperitoneal fat, epididymal fat and subcutaneous fat depot weights as independent predictors). With respect to glucose homeostasis and oxidative stress biomarkers we included the downstream effects of restriction on body composition and circulating hormone levels as predictors in the regression models. Type III sums of squares were used in all models. This analytical approach makes the assumption that the predictor variables are independent, and this assumption was clearly violated in our data, since the downstream body composition changes, resulting in particular from CR, were co-ordinated across the different tissues and correlated with each other [18]. Nevertheless, this analysis provides a first tentative step towards understanding the potential causal relationships that occur downstream of the initial experimental manipulation of restriction level.
